# Why Henry III of Navarre’s Hair Probably did not Turn White Overnight

**DOI:** 10.4103/0974-7753.66903

**Published:** 2010

**Authors:** Alexander A Navarini, Ralph M Trüeb

**Affiliations:** 1 Department of Dermatology, University Hospital of Zurich, Switzerland; 2 Skin and Hair Center, Zurich-Wallisellen, Switzerland

**Keywords:** *Canities subita*, henry III of navarre, saint bartholomew’s day massacre

## Abstract

Although a rare event, sudden whitening of hair (*canities subita*) has reportedly affected a number of well-known historical figures, usually in relation to dramatic events in their lives. Although early accounts are substantiated by more recent case reports in scientific literature, we suspect that the phenomenon is not only used as a literary means in fiction, with the aim of dramatizing, but probably also in historical accounts. For this purpose, we examine the case history of Henry III of Navarre who allegedly turned white on the evening of the Saint Bartholomew’s day massacre, and challenge this claim, due to inconsistencies in his biography, with the current pathophysiological understanding of *canities subita*.

## INTRODUCTION

Danger, long travel, want and woe,

Soon change the form that best we know;

For deadly fear can time outgo,

And blanch at once the hair.

(Marmion by Walter Scott)

History accounts that Henry III of Navarre’s hair (later Henry IV of France) allegedly turned white overnight on the occasion of St. Bartholomew’s day massacre. However, we would like to challenge this interpretation due to inconsistencies in his biography with the current pathophysiological understanding of *canities subita*.

**Figure 1:**
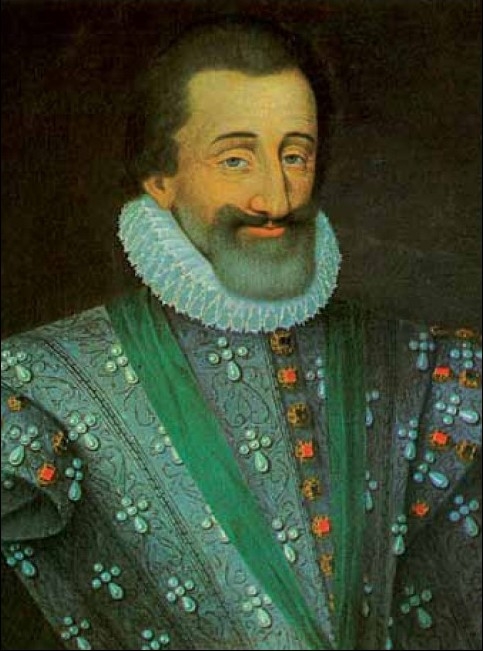
Portrait of Henry III of France[1]

**Figure 2:**
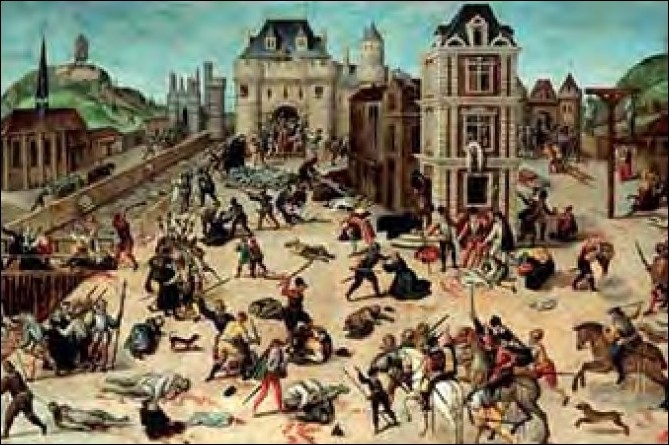
The Saint Bartholomew’s Day Massacre, François Dubois (1529–1584), painted ca. 1572–1584, oil on wood, current location: Musée cantonal des Beaux-Arts Lausanne, Switzerland

Henry III of Navarre [Figure 1] was born December 13, 1553, and baptized as a Roman Catholic, but raised as a Protestant by his mother, as she declared Calvinism the religion of Navarre. As a teenager, he joined his uncle Bourbon-Condé, the charismatic commander of the protestant Huguenot forces, in two campaigns in the French wars of religion. It had been arranged that to increase his political standing and influence, Henry would marry Marguerite de Valois (popularly known as Reine Margot), daughter of Henry II and Catherine de Medici. The celebrated wedding took place in Paris on August 19, 1572. On the occasion of the wedding, many of the most wealthy and prominent Huguenots had gathered in the largely Catholic Paris, where they were at the mercy of the adverse force. On the instigation of King Charles IV, selected Huguenot leaders were murdered on the eve of the Feast of Saint Bartholomew the Apostle on August 23, 1572 [Figure 2]. These assassinations were followed by a wave of Roman Catholic mob violence that spread throughout Paris and the rest of France, lasting for several weeks. Estimates for the number of casualties varied between 5,000 and 30,000 in total. Henry narrowly escaped death, thanks to the help from his wife Marguerite, although this marriage would remain unhappy. He was made to live at the court of France. Eventually he managed to escape in 1578, abjured Catholicism and and rejoined the Protestant forces, only to later permanently renounce Protestantism, with the encouragement of the great love of his life, Gabrielle d’Estrées, declaring that, “Paris was well worth a mass” (*Paris vaut bien une messe*). Moreover, this earned him the resentment of the Huguenots and of his former ally, Queen Elisabeth I of England, his entrance into the Roman Catholic Church secured him the succession to the French crown, and he was incarnated King of France on February 27, 1594.[1]

Among others, A.C. Lorry describes in “Tractus de Morbus Cutaneus” (1777), the overnight blanching of the hair of Henry of Navarre.[2] Perry, however, claims that it was exceeding grief that caused his mustache to whiten in the course of a few hours.[3] Xavier holds yet another version, in which Henry, troubled by the prospects of war, put his head in this hands to meditate on his sorrows, and when he lifted his face his mustache had grown white.[4] Similarly relating to the conflict between Roman Catholics and Protestants, Conrad Ferdinand Meyer (1825–1898) describes in his poem “Die Füsse im Feuer” the same phenomenon, on the occasion of the visit of a royal courtier who had tortured to death the wife of his host some years earlier. The Huguenot nobleman hosts the man without killing him despite ample opportunities.[5]

Again relating to a historical event, [Table 1] the phenomenon of the sudden whitening of the hair or canities subita is popularly also referred to as the Marie Antoinette syndrome,[6] named after the ill-fated queen of France whose hair blanched overnight the day before her last walk to the guillotine during the French Revolution. More recently, the term Thomas More syndrome has been proposed for men afflicted by the same disorder, as the English Martyr Saint Thomas More’s (1478–1535) hair is also said to have turned white the night before he was beheaded in the London Tower, however, the term Marie Antoinette syndrome for women has been retained.[7]

**Table 1:** Historical reports of sudden whitening of hair

Talmud scholar	83 AD[11]
Sir Thomas more	1535[7]
Marie antoinette	1793[6]

Canities subita, the Marie Antoinette, and the Thomas More syndrome are today understood to represent an uncommon variant of diffuse Alopecia Areata, where, in the naturally graying hair the pigmented hairs are preferentially targeted by autoimmunity. The sudden and selective loss of pigmented hairs within days gives the impression of sudden whitening of hair.[8,9] This has been challenged by some authors because some of the afflicted individuals, including our own published illustrations,[6] do not have conspicuously less hair following the event of sudden graying. To finally resolve this question, the hair density would have to be measured in an objective manner before and after the sudden whitening of hair.

According to the current, prevalent pathogenetic explanation, [Table 2] a pre-requisite for overnight whitening of hair would be that the individual in question has a salt-and-pepper pattern, with pigmented hairs interspersed with white hairs. Only if this is the case, can the pigmented hairs be selectively shed while the white hairs remain giving the impression of sudden whitening - otherwise total alopecia would result.

**Table 2:** Proposed pathophysiological mechanisms

Alopecia Areata diffusa with selective loss of pigmented hairs[8,9]
Sudden depigmentation along the hair axis[10]

Given that the current hypothesis of the syndrome’s pathophysiology holds true, it seems quite improbable that Henry III of Navarre’s hair should have turned white so rapidly, as he was only 19 years old at the time in question. He would have had premature canities, by definition hair graying before the age of 20, a condition usually transmitted in an autosomal dominant fashion and rather uncommon. Nevertheless, there is yet another theory as to how canities subita could occur that has not been addressed scientifically. When French neurologist Brown-Séquard observed white hairs in his beard he systematically plucked them as soon as they appeared. However, by the next day he again found white hairs of the same length.[10] His conclusion was that hair can change color along the axis of the hair, a proposition that has never been tested or challenged. Fitting to the theory, it is common to observe exclusively white next to pigmented hairs, but rare to observe hair pigmented on the distal end, while white on the proximal shaft during the physiological graying process – something that one would assume to be common at a certain age. Thus, the mystery of rapid whitening of hair is not fully revealed even in today’s harsh light of modern pathophysiological understanding.

Taken together, we argue that the historical accounts of sudden or overnight hair whitening should be taken with a grain of salt and questioned closely, as this phenomenon has not only been used as a literary stylistic means in fiction, with the aim to dramatize, but also in historical accounts with the claim of authenticity. It seems reasonable that sometime during his life, crowded with unpleasant incidents, Henry indeed became white-haired, but probably not on the night of Saint Bartholomew’s day in 1572.
